# Juxtaposition of bone age and sexual maturity rating of the Taiwanese population

**DOI:** 10.37796/2211-8039.1466

**Published:** 2024-12-01

**Authors:** Wen-Li Lu, Chung-Hsing Wang, Yi-Chun Lin, Fuu-Jen Tsai

**Affiliations:** a Department of Clinical Pathology, Chi Mei Medical Center, Tainan, Taiwan; b Department of Genetics, Endocrinology and Metabolism, China Medical University Children’s Hospital, Taichung, Taiwan; c School of Medicine, China Medical University, Taichung, Taiwan; d Department of Chinese Medicine, China Medical University Hospital, Taichung, Taiwan; e Department of Medical Research and Medical Genetics, China Medical University Hospital, Taichung, Taiwan; f School of Chinese Medicine, China Medical University, Taichung, Taiwan

**Keywords:** Bone age, Sexual maturity rating, Tanner staging, Pubertal onset, Puberty assessment

## Abstract

**Background:**

Bone age (BA) and sexual maturity rating (SMR) are crucial measures in assessing adolescent growth and development. However, studies specifically focusing on the association between BA and SMR in the Taiwanese adolescent population are limited. This study aims to utilize AI-assessed BA results to establish a relationship between BA and SMR in the Taiwanese adolescent population, particularly regarding the initiation of puberty.

**Materials and methods:**

The electronic medical records of bone age assessments conducted between January 1, 2019, and December 31, 2019, were reviewed retrospectively. For individuals with multiple records, only the latest entry within this period was retained. Records lacking a valid SMR or presenting significant medical conditions were excluded from the analysis. Males aged 7–17 years and females aged 6–16 years were included in the study.

**Results:**

The onset of puberty was observed to occur at a median bone age of 11.50 years (95% CI: 11.42–11.83) for males and 9.33 years (95% CI: 9.25–9.50) for females.

**Conclusion:**

The consistency between BA and SMR could serve as an alternative approach for assessing pubertal status in peripubertal children, providing a less intrusive evaluation regardless of chronological age (CA).

## 1. Introduction

Puberty is the period during which an individual matures to become capable of sexual reproduction. In the day-to-day practice of pediatric endocrinologists in Taiwan, there is an increasing number of outpatient visits involving the assessment of precocious puberty in both sexes [1]. Precocious puberty is generally defined as the development of secondary sexual characteristics before the chronological age (CA) of 8 years in females and 9 years in males. The evaluation of precocious puberty encompasses sexual maturity rating (SMR), bone age (BA), and hormone levels, with the rise in hormones providing verification for initiation of puberty.

SMR, also known as Tanner staging, is a system developed by Marshall and Tanner to classify changes in secondary sexual characteristics [2,3]. It involves visual and/or tactile assessments of two physical areas, arranged in increasing degree of maturity. Each assessment is rated on a scale from stage 1 to stage 5; stage 1 indicates the prepubertal state (absence of pubertal onset), and stage 5 corresponds to the final adult state. In males, SMR includes a stage of genital development and a stage of pubic hair development. In females, SMR incorporates a stage of breast development and a stage of pubic hair development. While the distinct characteristics of each stage were defined by Marshall and Tanner, an experienced professional is required to provide a precise rating.

BA is an indicator of physiological age as determined by skeletal maturation, interpreted from hand and wrist radiographs of the examinee [4]. Often, patients and families prefer SMR and BA examinations as they are the least invasive assessments, and their results may be produced instantaneously, unlike hormone level evaluations, which require days to process. While Marshall and Tanner associated SMR with the CA at which each stage was reached, increasing evidence suggests variability in the CA at which SMR is reached between individuals of different ethnicities [5]. Moreover, although the variability of BA was shown to resemble that of CA at pubertal onset [6], studies correlating SMR with BA are scarce.

To better assess the timing of pubertal onset with the least intrusive results, the relationship between SMR and BA should be explored to establish a more objective standard for providing care to these peripubertal children. In this study, we aim to establish an association between the SMR and BA of the Taiwanese population.

## 2. Methods

The electronic medical records (EMRs) of bone age assessments conducted between January 1, 2019, and December 31, 2019, were retrieved. A random serial number was assigned to each individual by the Big Data Center of China Medical University Hospital (CMUH-BDC) to ensure the anonymity of the individuals. The records were matched with the associated AI-assessed bone age (BA) results by the CMUH-BDC. The automated BA results were produced by the AI system trained to provide skeletal maturation assessment in accordance with the Greulich and Pyle method, with a performance consistent with that of qualified physicians [7,8]. The study was approved by the China Medical University & Hospital Research Ethics Center (CMUH110-REC1-040).

To increase reliability, EMRs of bone age assessments without concurrent SMR notes were removed during data cleansing. Since the evaluation of SMRs requires extensive experience, only medical practitioners who have undergone central training document SMRs in the EMRs. Medical practitioners who have not undergone central training refer clients needing such evaluations to pediatric endocrinologists in general terms. The central training involves at least 3 months of training with pediatric endocrinologists in the outpatient department at China Medical University Children’s Hospital.

To avoid repeated sampling, records of the same individual, i.e., with the same serial number, retained only the entry dated closest to the most recent BA assessment. Records without valid SMR documentation were removed. To establish an association between BA and SMR regarding the pubertal onset of the Taiwanese population, records with a prior medical history or past/current treatment using growth hormone therapies or gonadotrophin-releasing hormone agonists were excluded. Only males aged between 7 and 17 years and females aged between 6 and 16 years were included. Box-whisker plots were generated using the Tukey method, and the 95% confidence intervals (CI) of the median were calculated using Prism 9 (version 9.5.1).

## 3. Results

Between January 1, 2019, and December 31, 2019, 16,094 electronic medical records were identified at China Medical University Hospital containing information on bone age (BA). After excluding 7399 records of repeated prior visits and 3304 records lacking valid SMR documentation, 5391 records with both BA and SMR data from single individuals were obtained. Subsequently, 2364 records indicating medical history of disease status or past/current treatment using growth hormone therapy or gonadotropin releasing hormone agonists were excluded. After restricting the sample to males aged 7–17 years and females aged 6–16 years, a total of 2436 individuals were enrolled in this study; 861 were males and 1575 were females.

**Fig. 1:**
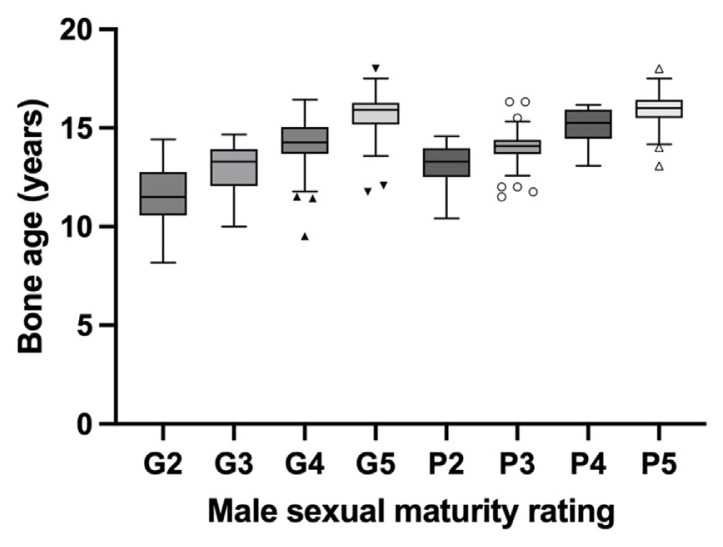
Bone age vs. male sexual maturity rating.

**Fig. 2:**
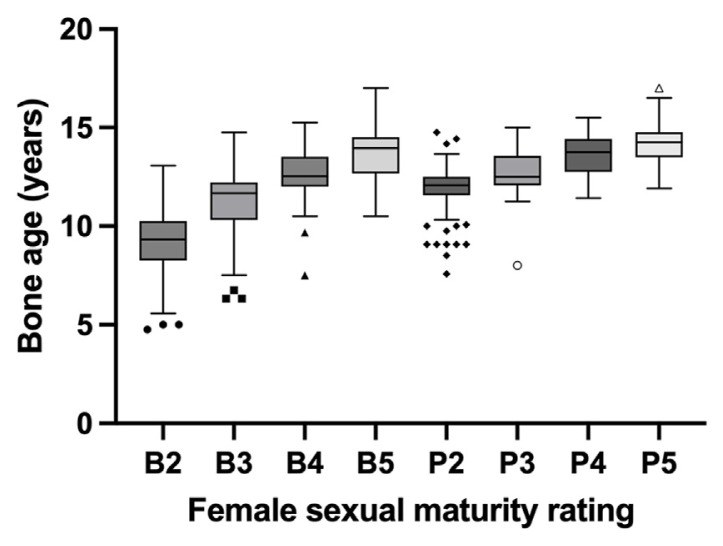
Bone age vs. female sexual maturity rating.

The pubertal onset for males was observed to occur at a median BA of 11.50 years (95% CI: 11.42–11.83), coinciding with genital development stages (Table 1 and Fig. 1). Furthermore, the BAs for successive presentations of both genital and pubic hair development in males exhibit distinct characteristics, as indicated by non-overlapping confidence intervals for both staging types. In females, pubertal onset was observed at a median BA of 9.33 years (95% CI: 9.25–9.50) concurrent with breast development (Table 2 and Fig. 2). Unlike males, the confidence intervals for female BAs overlapped between pubic hair stages 4 and 5, suggesting potential challenges in clinical differentiation at these stages.

## 4. Discussion

Although studies correlating skeletal maturity rating (SMR) to bone age (BA) are lacking in Taiwan, the peripubertal population there requires evaluation for precocious puberty. Literature indicates variability in BA determination across ethnicities. Specifically, Asian males, including the Taiwanese population, have been advised against using the Greulich and Pyle method for BA assessment [9]. Considering the BA discrepancy in Taiwanese children was reported by Yuh et al. [10], an alternative approach may be warranted when assessing puberty in the Taiwanese population.

In this study, we aimed to assess BA in relation to SMR for the initiation of puberty among Taiwanese individuals, aiming for a minimally intrusive approach. We found non-overlapping confidence intervals for BA across sequential SMR stages except for female pubic hair stages 4 and 5. Lower confidence limits could serve as cut-off values prompting further investigation, including blood-work, in cases of inconsistency. Therefore, irrespective of chronological age (CA), close monitoring of puberty progression is advised for males with BA exceeding 11.50 years or females with BA exceeding 9.33 years, even in the absence of secondary sexual characteristics.

Etiologic investigation is crucial when discrepancies between BA and SMR are observed in pediatric patients. BA assessment considers secondary ossification and growth plate fusion, influenced by factors such as growth hormones, insulin-like growth factor-1, thyroid hormone, estrogen, androgen, proteoglycan, and parathyroid hormone-related peptide [11]. On the other hand, SMR assessment involves the development of breasts, testes, penises, and pubic hair, regulated by hormones such as estrogen, growth hormone, progesterone, gonadotropin-releasing hormone, follicle-stimulating hormone, luteinizing hormone, and testosterone during puberty [12,13]. Assessment of inconsistency between bone age (BA) and skeletal maturation rate (SMR) would assist in evaluating disturbance involving these factors. For instance, individuals with Turner syndrome have shown to display a progressive delay in BA up to 2 years by the age of 16 [14], possibly due to epiphyseal plate closure failure, while exhibiting normal pubic hair development. It is indispensable to identify the chromosomal anomaly after acknowledging the inconsistency to guide appropriate treatment interventions. Conversely, individuals with obesity were found to present with advanced BA despite a lower prevalence of pubarche, menarche, and voice breaking [15,16]. It remains essential to conduct the necessary workup and management considering the burden of obesity and its associated complications [17].

Several limitations are inherent in this study. Firstly, the sample size could have been larger if SMR had been consistently recorded, underscoring the critical role of proper healthcare documentation. However, to mitigate potential rater effects, we employed a matched-records approach between concurrent bone age (BA) examinations and SMR notes within electronic medical records (EMRs). Secondly, despite stringent inclusion criteria, outliers were identified; however, their impact is expected to be minimal given that medians are less susceptible to distortion compared to means. Finally, further investigations within the Taiwanese population are indispensable to accurately delineate genital stages 4 and 5 among Taiwanese girls, enhancing the representation of female adolescents at the culmination of puberty.

In summary, BA and SMR assessments are vital in evaluating puberty, particularly in populations like Taiwanese children where BA discrepancies have been noted. Aligning BA with SMR offers a less invasive alternative for assessing pubertal progression, supported by identified BA cut-off values for each SMR stage, guiding clinical decision-making regarding further diagnostic workup.

## Figures and Tables

**Table 1:** 95% Confidence intervals of medians in bone age (years) of males.

SMR	Number of subjects	Median	Lower confidence limit	Upper confidence limit	Actual confidence level
G2	187	11.50	11.42	11.83	95.97%
G3	166	13.29	13.00	13.58	96.42%
G4	130	14.25	14.08	14.50	95.67%
G5	154	15.92	15.75	16.00	95.64%
P2	104	13.29	13.00	13.58	96.10%
P3	80	14.08	14.00	14.17	96.70%
P4	69	15.25	14.83	15.58	97.05%
P5	127	16.00	15.83	16.08	96.72%

G, genital staging; P, pubic hair staging.

**Table 2:** 95% Confidence intervals of medians in bone age (years) of females.

SMR	Number of subjects	Median	Lower confidence limit	Upper confidence limit	Actual confidence level
B2	751	9.33	9.25	9.50	95.13%
B3	320	11.67	11.50	11.75	96.16%
B4	110	12.54	12.25	12.75	95.52%
B5	150	13.96	13.75	14.25	95.91%
P2	142	12.08	11.92	12.25	96.45%
P3	107	12.50	12.33	12.75	96.71%
P4	39	13.75	12.92	14.25	97.63%
P5	106	14.25	14.00	14.42	95.91%

B, breast staging; P, pubic hair staging.
